# Genotyping of *Clostridium perfringens* Isolates from Domestic Livestock in Saudi Arabia

**DOI:** 10.1155/2020/9035341

**Published:** 2020-03-23

**Authors:** Sawsan A. Omer, Ebtesam M. Al-Olayan, Salah Eldin H. Babiker, Mohammed Z. Aljulaifi, Abdulaziz N. Alagaili, Osama B. Mohammed

**Affiliations:** ^1^Department of Zoology, College of Science, King Saud University, University Centre for Women Students, P. O. Box 22452, Riyadh 11495, Saudi Arabia; ^2^Department of Veterinary Laboratories, Ministry of Agriculture, King Saud University, Riyadh, Saudi Arabia; ^3^KSU Mammals Research Chair, Department of Zoology, College of Science, King Saud University, P.O. Box 2455, Riyadh 11451, Saudi Arabia

## Abstract

The present study was undertaken to confirm the genetic identity of *Clostridium perfringens* isolates from domestic livestock in Saudi Arabia and to characterize the genes encoding to alpha, beta, epsilon, and iota (*α*-, *β*-, *ε*-, and *ι*-) toxins. *C. perfringens* were confirmed in 104 out of 136 isolates on multiplex polymerase chain reaction using specific primers amplifying genes related to toxins produced by *C. perfringens*. Genes encoding *α*-toxins were detected in 104 samples. Of the isolates, 80.8% were diagnosed as type A, 15.4% as type D, 2.9% as type C, and 0.96% as type B. None of the isolates has genes encoding iota (*ι*-) toxin. All isolates investigated yielded enterotoxin (*cpe*) products and none yielded *β*2 (cpb2-toxin) or *NetB* products. *PLC* gene sequences encoding *α*-toxin showed >96.7% similarity. Isolates which had *α*-toxins as well as enterotoxin (*cpe*) are regarded as type F. Phylogenetic analysis using maximum likelihood analysis yielded two clades, and the majority of the isolates were in one group while only two isolates clustered on the second clade. Within the Kingdom of Saudi Arabia strains, 54 variable positions and 23 polymorphic amino acids were noticed. Isolates with *ε*- and *β*-toxins were variable and were found to be close to those published for *C. perfringens*. *ETX* gene sequences encoding *ε*-toxins were found to be related to *CPE* sequences.

## 1. Introduction

The anaerobe and spore-forming *Clostridium perfringens* is a Gram-positive rod-shaped bacterium detected in soil and water as well as among intestinal bacterial flora of animals and man [1]. It is categorized with the most widespread bacteria, which occurs in different environments such as soil, sewage, food, feces, and the normal intestinal flora of humans and animals [2]. The toxins produced by different species of *Clostridium* are more than those produced by other eubacteria [3]. All strains of *C. perfringens* possess the gene encoding for phospholipase C (*plc*) (also referred to as alpha toxin *α*) in combination with differential expression of 3 major toxin-encoding genes (beta *β*, epsilon *ε*, and iota *ι*) used to classify strains as toxinotypes A to E [4]. *C. perfringens* isolates from type A produce only *α*-toxin, those from type B produce *α*-, *β*-, and *ε*-toxins, those from type C secrete *α*- and *β*-toxins, those from type D produce *α*- and *ε*-toxins, and bacteria from type E produce *α*- and *ι*-toxins [5]. *C. perfringens* is a human and livestock pathogen, resulting in disease in different tissues as well as intestinal diseases in the form of enterotoxaemia. *C. perfringens* type A toxigenic group causes food poisoning in humans and enteritis in domestic and wildlife animal species, type B and C cause necrotizing enteritis and enterotoxaemia in cattle, sheep, and other animals, type D affects sheep, goats, and cattle mainly, and type E causes enteritis in rabbits, sheep, and cattle [6, 7]. Recent results by Rood and others suggested a modification for the toxinotyping of *C. perfringens* which required the inclusion of the enterotoxin (*CPE*) and NetB toxins produced by the bacterium [8]. They proposed that strains which produce *α*-toxin as well as *CPE* are assigned type F whereas those producing *α*-toxin and NetB toxins are assigned type G. In both cases, no other toxins such as *β*-, *ε*-, and *ι*-toxins are produced.

The prevalence of *C. perfringens* in Saudi Arabia was studied by some investigators [9, 10]. In 2006, during the annual Hajj pilgrimage, a group of Saudi soldiers were diagnosed for having gastroenteritis following a rice lunch contaminated with *Bacillus cereus* and *C. perfringens* [9]. Moussa and Hessan [10] detected *C. perfringens* in 20% of the calves, sheep, and poultry investigated. *C. perfringens* was detected in the dromedary camel in the eastern region of Saudi Arabia, where a prevalence of 23.4% was reported from the samples investigated [11].

Diagnosis of *C. perfringens* enterotoxaemia in various animal species is dependent on clinical signs as well as pathological findings; however, characterization of toxins in intestinal contents is of importance to confirm the clinical diagnosis.

The widely used identification of the toxins is based on mice neutralization test, but this method is expensive, time-consuming, and subjecting laboratory animals to stress and unnecessary risk of being affected by disease [12]. Enzyme-linked immunosorbent assay (ELISA) kits have also been used for the detection of clostridial toxins [13, 14]. DNA-based techniques, such as the polymerase chain reaction (PCR) was developed for *C. perfringens* genotyping which is a reliable alternative method to replace testing in laboratory animals. Different PCR protocols were employed to genotype *C. perfringens* isolates with respect to the genes *cpa*, *cpb*, *etx*, *iap*, *cpe*, *NetB*, and *cpb2*, which encode the *α*-, *β*-, *ε*-, *ι*-toxins, *CPE*, NetB, and *β*2-toxins, respectively [4, 15].

In the present study, *C. perfringens* isolated from livestock in Saudi Arabia has been characterized and the sequence variation in *α*-, *ε*-, and *β*-toxins producing isolates has been investigated.

## 2. Materials and Methods

Isolates of *C. perfringens* were obtained from field investigations for the diagnosis of enterotoxaemia in domestic livestock in Saudi Arabia. Intestinal swabs and intestinal contents were taken to the laboratory of the Ministry of Environment, Water and Agriculture (MEWA), KSA. In the laboratory, samples were subjected to microscopic examination using Gram stain as well as to serological testing. Representative isolates were subjected to DNA extraction, polymerase chain reaction, and DNA sequencing.

### 2.1. Microscopic Examination of Gram-Stained Smears

Impression smears were prepared from parts of the small intestines and smears were also prepared from suspected bacterial isolates. Smears were air-dried, heat fixed, stained with Gram stain, and examined microscopically for detection of large-sized Gram-positive bacilli similar to clostridia.

### 2.2. Detection and Typing of *C. perfringens* by ELISA Assay

The Bio-X enterotoxaemia ELISA kit (Bio-X Diagnostics, Belgium) was used to detect *α*-, *β*-, and *ε*-toxins, cellular antigens of *C. perfringens* in clinical samples, and bacterial cultures according to the manufacturer's instructions. Typing of *C. perfringens* isolates into the different types A, B, C, or D was done by comparing the types of toxins detected in the clinical material or culture supernatants of pure colonies of *C. perfringens* isolates grown in the liquid Trypticase Glucose Yeast (TGY) extract medium. The TGY medium is composed of Tryptone, yeast extract, glucose, K_2_HPO_4_, agar, and water.

### 2.3. Bacterial DNA Extraction, Polymerase Chain Reaction (PCR), DNA Sequencing, and Phylogenetic Analyses

DNA was extracted from 136 supposedly *C. perfringens* isolates based on the microscopic examination, using the QuickExtract™ Bacterial DNA Kit from Epicentre**®** (an Illumina® Company). Polymerase chain reaction (PCR) and multiplex PCR were performed using the primers illustrated in Table 1. The *plc* gene was PCR amplified from extracted DNA or cDNA using the primer pair designed by Baums and others [15]. Amplifying *NetB* gene was performed using primers APK78-F and APK79-R as indicated by Keyburn and others [16].

The *plc* (*cpa*), *cpb*, *ext*, *ipa*, *cpe*, *NetB*, and *cpb2* genes were amplified in 25 *μ*l volumes containing 5 *μ*l PCR buffer, 1 pmol each specific primer, 200 mM each dNTP, 4 U MyTaq DNA polymerase (Bioline, London, UK), and 2 *μ*l of DNA extract. Thermal cycling conditions were as follows: 2 min at 94°C, followed by 35 cycles of 94°C for 30 sec, 55°C (up to 58°C depending on the primer) for 30 sec, 72°C for 30 sec, and a final extension at 72°C for 5 min. The PCR products were visualized using stained ethidium bromide 1.5% agarose gel exposed to ultraviolet light, and digital images were taken from the PCR product through gel documentation system. Positive PCR products obtained were purified using the QIAquick kit (QIAGEN, Hilden, Germany) according to the manufacturer's instructions.

Purified PCR products were sequenced using the Macrogen facility, Seoul, South Korea. The *plc* gene sequences were manually aligned according to their nucleotides and the deduced amino acid sequences using MEGA X, and all alignments were refined by manual inspection.

Phylogenetic analyses were conducted using the computer program MEGA X [17]. The best-fit substitution model was TN93+G+I. The statistical reliability of internal branches was assessed from 1000 bootstrap pseudoreplicates. Phylogenetic trees were generated in MEGA X using the maximum likelihood (ML) approach.

The deduced amino acids from the coding regions were obtained using MEGA X. Then, they were compared with the existing sequences in the NCBI Protein database to identify homologous sequences through PSI-BLAST. Sequences from different strains were aligned with the ClustalW program using the MAFFT, a multiple sequence alignment program [18]. The secondary structure of *plc* (*α*-helix and *β*-sheet) was predicted using the PDBSAS server [19]. Relevant annotations were mapped using a consensus sequence recovered from the PDB server. Phylogenetic trees were generated using the maximum likelihood (ML) analysis in MEGA X.

### 2.4. Nucleotide Sequence Accession Numbers

The *plc* gene sequences (*α*-toxins) have been deposited in the GenBank database under accession numbers (48 sequences) MN646319 to MN646366. *etx* gene sequences (13 sequences) accession numbers are from MN649858 to MN649870, *cpb* gene sequences (4 sequences) accession numbers are from MN683524 to MN68352527, and *cpe* gene sequences (40 sequences) are from MN683528 to MN683567.

## 3. Results

Clinical samples collected for bacteriological identification yielded *C. perfringens* based on cultural, microscopic, and cellular antigenic characterizations.

Molecular characterization of 136 isolates showed that 104 isolates yielded positive PCR products. Of those 138 isolates, 84 (80.8%) had only the *plc* gene (type A), 16 (15.4%) isolates had the *etx* gene as well as the *plc* gene (type D), 3 (2.9%) isolates had the *cpb* gene as well as the *plc* gene (type C), and 1 (0.96%) isolate had the *plc*, *cpb*, and *etx* genes (type B). All isolates investigated in the present study had the *cpe* gene; however, none had *iap*, *cpb2*, or *NetB* genes.

**Figure 1:**
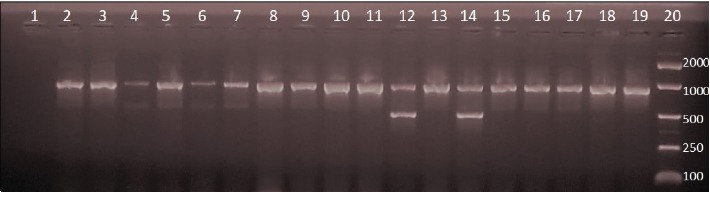
A multiplex PCR typing of selected *Clostridium perfringens* isolates. Lane 1: empty lane, all the 18 isolates showed the PCR product resulting from amplification of *plc* (*cpa*) gene showing a fragment size of 900 bp. Lanes 4-7 produced *etx* gene (a fragment size of (396 bp) in addition to *plc* gene. Lanes 12 and 14 produced *cpb* gene (a fragment size of 612 bp) in addition to the *plc* gene. Lanes 2, 3, 8-11, 13, 15-19 produced only the *plc* gene. Lane 20: Bioline EasyLadder 1.

Figure 1 shows representative isolates which showed positive PCR products in a multiplex PCR. The expected sizes of the PCR product from different primers are shown from representative samples.

### 3.1. Alpha (*α*-*plc*) Gene Characterization

A total of 48 sequences from alpha toxins were analyzed. Sequences of alpha toxins obtained in the present study were highly similar, and they showed a similarity of >96.7%. There was no specific sequence type associated with a known location. The sequences investigated were from 5 different cities including Riyadh, Hofuf, Jeddah, Taif, and Qassim. The dataset used in the analysis included 104 sequences. Of those, 48 were new sequences (present study), 22 were published from animals and humans in the USA [20], 32 strains were isolated from Danish broiler chickens grouped according to pulsed-field gel electrophoresis (PFGE) profiles [21], one unpublished sequence (MK599266) was from camel, and one was out-group *Clostridium novyi* (D32125).

**Figure 2:**
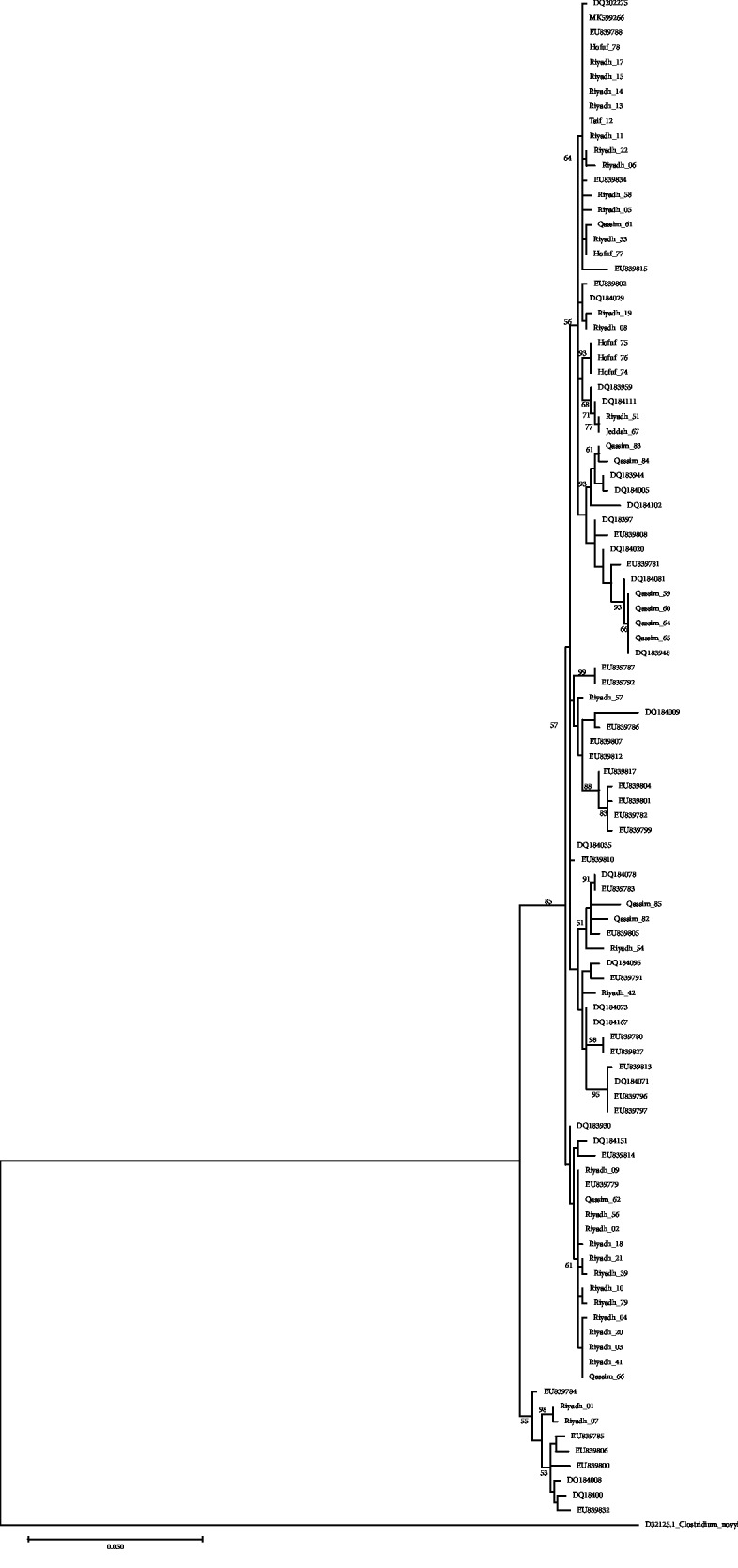
Maximum likelihood phylogenetic tree showing the relationship of the *plc* gene from 48 isolates reported in the present study with sequences from the GenBank. The tree is drawn to scale, with branch lengths measured in the number of substitutions per site. Only significant bootstrap values are shown. This analysis involved 102 nucleotide sequences. There was a total of 842 positions in the final dataset. Evolutionary analyses were performed in MEGA X.

The ML topology showed two major clades with high bootstrap values, the first is divided into 10 subclades including 46 of the isolates. The most divergent isolates, 1 and 7, were localized in the second clade (Figure 2). The strain MK599266 grouped with strains from Hofuf and Riyadh.

**Figure 3:**
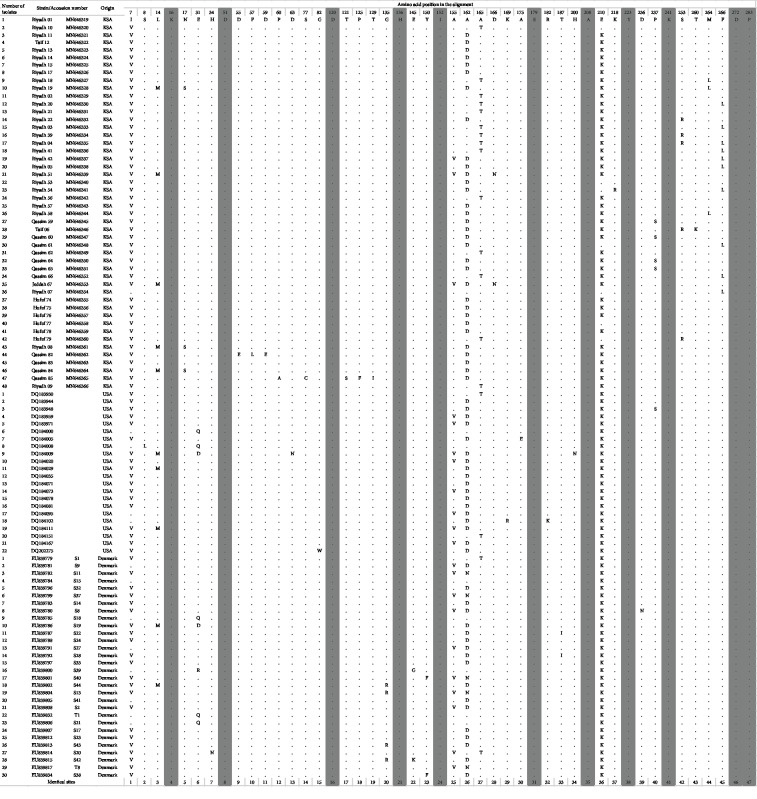
Variable amino acid positions of the defined alpha toxin sequence types from USA, Denmark, and KSA (this study). Identical amino acids sites are shaded.

The *plc* gene was reported to be highly conserved in *C. perfringens* strains. We observed 275 and 47 variable positions, respectively, out of the 860 nucleotides and 272 deduced amino acids. The studied protein-coding region displayed moderate-level nucleotide diversity (Pi = 0.019). The dataset generated 74 haplotypes characterized by a high haplotype diversity (Hd = 0.987). Within KSA samples, we observed 54 variable positions and 23 polymorphic amino acids (Figure 3).

*α*-Gene sequences obtained in the present study were compared to similar sequences obtained from the GenBank. The sequences from the Kingdom of Saudi Arabia were scattered and clustered with sequences obtained from Denmark and USA (Figure 2). Fourteen sequences were clustered in one group with some other sequences from the GenBank while other sequences were scattered between other sequences. Protein sequences differed in 22 positions between isolates from Saudi Arabia, and they showed 31 different types. They showed differences in 41 amino acid position when compared with isolates from USA and Denmark; however, some of them have shown similarities (Figure 3). The sequence variation was not related to the pathogenicity of the strain as all organisms investigated in this study were obtained from pathological cases.

**Figure 4:**
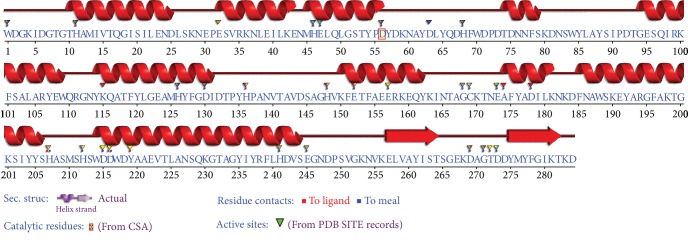
Alpha toxin sequence annotation by structure SAS (sequence annotated by structure, EMBL-EBI) against all the proteins of known 3D structure in the Protein Data Bank (PDB code of the protein presenting the highest % of identity: 1qmd, 1qm6, 1sb4, and 1ca1).

The secondary structure prediction revealed that *plc* amino acid sequences of *α*-toxin mainly consists of alpha-helices (Figure 4). The *α*-toxin enzyme contains an *α*-helical N-terminal domain (residues 1–246) encompassing the active site and a *β*-sandwich C-terminal domain (residues 247–255). The *β*-sandwich domain includes a calcium-binding pocket (Figure 4). The Protein Data Bank (PDB) multiple alignment confirmed the structural composition and showed 9 proteins with identity percentage varying between 95.4% and 98.9%, all *α*-toxin belonging to *C. perfringens. Clostridium absonum α*-toxin showed only 62% identity compared to those obtained from *C. perfringens*.

### 3.2. Epsilon (*ε*-) Gene Characterization

**Figure 5:**
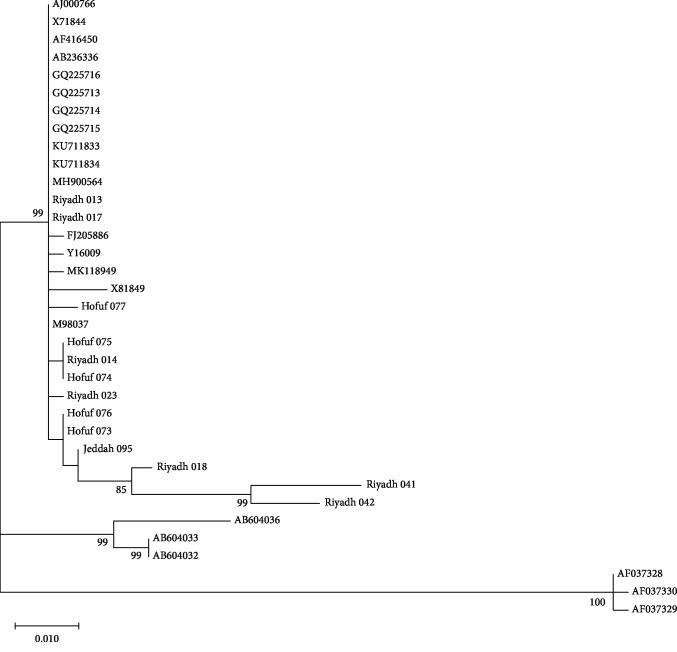
Maximum likelihood phylogenetic tree showing the relationship of the epsilon toxin-producing genes from 13 sequences reported from *C. perfringens* isolates in the present study with related sequences from the GenBank. The tree is drawn to scale, with branch lengths measured in the number of substitutions per site. Only significant bootstrap values are shown. This analysis involved 35 nucleotide sequences. There was a total of 438 positions in the final dataset. Evolutionary analyses were performed in MEGA X.

The dataset included 33 sequences of *ε*-gene, 13 new sequences (KSA) and 20 published previously (retail meat products, Japan; calves, lamb, human, and food, USA; and foal UK). We observed 69 variable sites and 48 were parsimony informative sites. We observed 32 variable positions out of the 146 deduced amino acids. The maximum likelihood (ML) tree generated revealed that *ε*-gene sequences from Saudi Arabia grouped with sequences obtained from *cpe* genes from different animal species and from humans (Figure 5).

### 3.3. Beta (*β*-) Gene Characterization

The dataset included 16 sequences comprising 4 new and 12 reference sequences published previously (HQ111476-82 and KU836730 from Iran, L13198 from the United Kingdom, and X83275 from Iceland). Annotation of the obtained sequence revealed that the region comprising between 63 and 68 corresponds to a regulatory class=“ribosome binding site.” The fragment from the position 76 to 560 corresponds to a partial product of the *β*-toxin. Multiple alignments of *cpb* gene sequences yielded 21 variable sites, and only three were in the coding region.

Homology of *β*-toxin with other proteins: the deduced partial *β*-toxin amino acid sequence was screened against the Swiss-Prot. A significant homology was found with the deduced amino acid sequence of *Staphylococcus aureus α*-toxin (sequence ID: P09616, 26% identities, 47% positives substitutions), *ϒ*-toxin component B (sequence ID: Q6GE12, 25% identities, 44% positives substitutions), and leukocidin-F (sequence ID: P31715, 25% identities, 44% positives substitutions). The sizes of these proteins vary between 319 and 325 amino acids.

### 3.4. Enterotoxin (*cpe*) Production

All isolates showed positive results for the *C. perfringens* enterotoxin (*cpe*). Different haplotypes were reported; however, the majority of the haplotypes were identical to those reported for *C. perfringens* available in the GenBank.

## 4. Discussion

The identity of *Clostridium perfringens* isolated from domestic livestock in Saudi Arabia has been confirmed using molecular techniques. Furthermore, different genes responsible of excreting each toxin have been identified and characterized. *C. perfringens* type A has been reported in 80.8% of the bacterial isolates, type D was reported in 15.4%, type C was isolated from 2.9%, and type B was reported in 0.98%. This finding was similar to a previous work which recorded the four types of *C. perfringens* reported in this study but with variable rates of prevalence [11]. They reported *C. perfringens* type A in 33.3%, type B in 5.3%, and types C and D both in 5.3% of the camel investigated. On an earlier study, *C. perfringens* types A in 81.5%, B in 3.7%, C in 3.7%, and D in 11.1% of sheep, goats, and chicken investigated in Riyadh region were reported [10]. In both studies, the neutralization test in mice were used and they did not include either *cpe* or *NetB* gene in their studies. Organisms which were recognized, in the present study, as type A may be designated as type F rather than type A as all produced *CPE* toxin together with *α*-toxin according to the suggestion made by Rood and others [8]. We were able to demonstrate the presence of *C. perfringens* type F for the first time in Saudi Arabia. Results of the present study indicate that *C. perfringens* type A (or type F) is the most prevalent type, and these results were also similar to those found in previous studies [22–27]. The gene coding to *α*-toxin in Saudi Arabia was found to show variability, and it is interesting that some of the strains are related to strains isolated from Japan and USA [20, 21]. Furthermore, strains which were reported from Riyadh clustered with those obtained from Taif, Jeddah, and Qassim. Such finding can be attributed to the fact that animal movement in Saudi Arabia is inevitable and Riyadh is a big market for livestock; therefore, animals are transported between different cities, and hence, bacterial infection can easily be found in various places.

Epsilon-coding gene sequences were found to be related to *cpe* genes produced by type C and D isolates from different animal species in the study conducted by some investigators [28, 29]. *CPE* loci were found to be divergent in *C. perfringens* isolates. All *cpe*-positive type D isolates studied previously were found to carry both *cpe* and *etx* genes on the same plasmid [28, 30]. It is likely the case of having *etx* grouped with *cpe* in the current study. Such observations may indicate that a similar genetic element found in the plasmid has mobilized the conserved *cpe* locus from a progenitor *cpe*-carrying plasmid found in type D isolates into other plasmids in the same isolate. This finding requires further investigations to clarify it.

In the present study, enterotoxin gene (*cpe*) was reported in all bacterial isolates while the *cbp2* gene was not detected in any of the isolates. Unlike what we found in our investigation, where *β*2-toxin was reported from all isolates, they were obtained from the dromedary camel [11]. *β*2-Toxin toxin-producing *C. perfringens* type A has been associated with disease in several animal species, such as piglets suffering from hemorrhagic enteritis in 1997, and it has been reported to possess a cytological effect on certain cell lines and is lethal to mice [31]. The *β*2-toxin is being associated mainly with type A but has also been identified in association with types C and E in piglets and calves, respectively [32]. Similar findings by other investigators have also indicated that none of the isolates studied was positive to specific sequences of the gene coding the enterotoxin production (*cpe*) [33, 34]. The enterotoxin (*cpe*) plays an important role in the development of intestinal disease in many animal species including man [7, 35]. Tight junction components between the intestinal cells which regulate the permeability of ions and macromolecules are included in the smaller and larger protein complexes which cause paracellular permeability changes [36, 37]. The mechanism of action of enterotoxin is binding to the intestinal epithelial cells forming a series of protein complexes in cell membranes, which ultimately results in altering membrane permeability and cell lysis. *β*2-Toxin was found to be present among *cpe-*positive *C. perfringens* type A isolates from humans with antibiotic-associated diarrhea. Of 35 *cpb2-*harbouring enterotoxigenic *C. perfringens* type A strains, production of *β*2-toxin was produced in 34 isolates *in vitro* [38]. The presence of *cpb2* was less common among *C. perfringens* strains isolated from humans suffering from food poisoning [38].

Genotypic differences exist between *cpe* type A-positive isolates causing food poisoning and those causing nonfoodborne gastrointestinal disease [39]. It has been demonstrated that most, if not all, *C. perfringens* type A food poisoning isolates carry their *cpe* gene on the chromosome while most, if not all, *CPE*-associated nonfoodborne human gastrointestinal diseases carry a plasmid *cpe* gene [40, 41]. *CPE* sequences reported in the present study showed high similarity to those described from the GenBank, and they are of plasmid origin. This suggested that the *cpe* sequences reported from all *α*-toxin-producing strains are probably of plasmid origin and not of chromosomal origin.

Overall, the mutations that were found in the amino acid sequences of *α*-toxin did not significantly alter the predicted structure of the proteins. The studied isolates of *plc* showed high sequence identity (>95%) to PDB *Clostridium* strain. None of the detected amino acid mutations occurred in the active site or in the partial C-terminal domain (Figure 2). These regions have been shown to play key roles in protein-membrane interactions prior to phospholipid cleavage [42].

## 5. Conclusion

*Clostridium perfringens* types A, B, C, and D have been reported from the Kingdom of Saudi Arabia. *C. perfringens* type A has been reported in 80.8% of the bacterial isolates, type D was reported in 15.4%, type C was isolated from 2.9%, and type B was reported in 0.98%. There was some genetic variation in the conserved *plc* gene which is responsible of the production of alpha toxins. In the present study, 275 and 47 variable positions, respectively, out of the 860 nucleotides and 272 deduced amino acids, were observed. All isolates studied produced *CPEs*. When considering vaccine production, field isolates which comprise all the strains must be considered.

## Figures and Tables

**Table 1:** Primers used for amplification of the genes responsible of producing specific toxins and the expected amplicon size together with the annealing temperature for the PCR.

Gene	Primer sequence 5′ to 3′	Amplicon size (bp)	Annealing temp (°C)
*Plc*, *cpa* (*α*-toxin)	CPA5L: AGTCTACGCTTGGGATGGAA CPA5R: TTTCCTGGGTTGTCCATTTC	900	55

*cpb* (*β*-toxin)	CPBL: TCCTTTCTTGAGGGAGGATAAA CPBR: TGAACCTCCTATTTTGTATCCCA	612	55

*etx* (*ε*-toxin)	CPETXL: GGGGAACCCTCAGTAGTTTCA CPETXR: ACCAGCTGGATTTGAGTTTAATG	396	55

*ipa* (*ι*-toxin)	CPIL: AAACGCATTAAAGCTCACACC CPIR: CTGCATACCCTGGAATGGCT	293	55

*Cpe* (CPE)	CPEF: GGAGATGGTTGGATATTAGG CPER: GGACCAGCAGTTGTAGATA	233	56

*cpb2* (*β*2-toxin)	CPBL: CAAGCAATTGGGGGAGTTTA CPBR: GCAGAATCAGGATTTTGACCA	200	55

NetB toxin	AKP78: GCTGGTGCTGGAATAAATGC AKP79: TCGCCATTGAGTAGTTTCCC	560	55-58

## Data Availability

The data used to support the findings of this study are available from the corresponding author upon request.
